# Statistical validation of surrogate outcome measures

**DOI:** 10.1186/1745-6215-12-S1-A93

**Published:** 2011-12-13

**Authors:** Marc Buyse

**Affiliations:** 1 IDDI, Louvain-la-Neuve, and Hasselt University, Diepenbeek, Belgium

## 

With the large number of promising new molecules that are currently available for clinical testing, clinical trials need to detect a drug’s benefit (or harm) as quickly as possible. In parallel with the need for speed in clinical development, advances in molecular biology, high throughput technologies and imaging techniques provide investigators with an ever growing number of biomarkers which can be used for a variety of purposes: to inform go / no go decisions in early clinical development, to stratify patients, to target subsets, to adjust therapy, or to replace clinical endpoints in the comparison of the new drug with standard treatments. This talk will focus on the latter goal, and will discuss the type of statistical evidence required for a biomarker (or a clinical endpoint) to be an acceptable outcome measure for use in clinical trials [1].

Historically, the first formal definition of surrogacy is due to Prentice [2]. While this definition has had a huge role in focusing attention on the need for formal statistical criteria to validate a potential surrogate, it may also have led to excessively pessimistic views about the potential for any outcome measure (whether it be a clinical endpoint or a biomarker) to ever qualify as a good (let alone “perfect”) surrogate. A large amount of research has been devoted to operational criteria to implement Prentice’s definition in practice.

An even larger amount of research has been devoted to a different approach based on statistical associations between the endpoints (surrogate and true), and between treatment effects on these endpoint. It has been proposed that a good surrogate must be tightly correlated with the true endpoint (the so-called “individual-level” association), and that the treatment effect on the surrogate must be tightly correlated with the treatment effect on the true endpoint (the so-called “trial-level” association) [3]. Showing that both criteria are met usually requires a meta-analysis of randomized trials, or one large trials that can be broken down in smaller units (such as participating countries). When such data are available, the predictive value of potential surrogate biomarkers can be investigated, and the “surrogate threshold effect” can be estimated as the minimum effect on the surrogate biomarker that predicts a statistically significant effect on the clinical endpoint [4].

A very different line of research has evolved from concepts of causal inference, in particular the concept of “principal stratification”, in which treatment effects on the true endpoint are estimated within strata defined by different surrogate values [5]. The conceptual elegance of this approach has not yet led to convincing applications, in large part because it has proven challenging to find good estimation methods for the counterfactual probabilities that are required to validate a surrogate [6]. It is likely, however, that causal inference will play a key role in future attempts to validate surrogate endpoints.
